# Women's mental health: What progress have we made?

**DOI:** 10.1192/j.eurpsy.2021.53

**Published:** 2021-08-13

**Authors:** H. Herrman

**Affiliations:** Centre For Youth Mental Health, University of Melbourne, Victoria, Australia

**Keywords:** perinatal health, women's mental health, violence

## Abstract

Significant progress is being made in strengthening perinatal mental health support systems and in several related areas of women’s mental health. Mental health support for women and families during pregnancy and the first year after birth – the perinatal period – remains a priority in most parts of the world. Mental disorders are among the most common perinatal health problems, with over 25% of women in many scarce resource countries and 10% in wealthy countries experiencing a disorder. There is growing recognition of feasible and effective ways to reduce the harm to women and children and their families through societal as well as health system initiatives. Successful initiatives including training and support for health workers and cross-sectoral work to prevent violence in families are operating in a number of countries. The presentation will consider how psychiatrists and other mental health professionals can contribute to the spread, scope and sustainability of this work, and other related contributions to women’s mental health including the prevention of violence in the family.

**Disclosure:**

No significant relationships.

